# Depth and type of substrate influence the ability of *Nasonia vitripennis* to locate a host

**DOI:** 10.1093/jis/14.1.58

**Published:** 2014-01-01

**Authors:** Christine Frederickx, Jessica Dekeirsschieter, François J. Verheggen, Eric Haubruge

**Affiliations:** Department of Functional and Evolutionary Entomology, Gembloux Agro-Bio Tech, University of Liege, Passage des Déportés 2, 5030 Gembloux, Belgium

**Keywords:** blow fly, burrowing behaviour, *Calliphora vicina*, Diptera, forensic entomology, parasitoid

## Abstract

The foraging behaviour of a parasitoid insect species includes the host’s habitat and subsequent location of the host. Habitats substrate, substrate moisture, and light levels can affect the host searching of different species of parasitoids. However, the depth at which parasitoids concentrate their search effort is another important ecological characteristic and plays an important role in locating a host. Here, we investigated the ability of a pupal parasitoid, *Nasonia vitripennis* Walker (Hymenoptera: Pteromalidae), to penetrate and kill fly pupae located at different depths of the substrate. Three different types of substrate were tested: loam soil, compost, and vermiculite substrate. In both loam soil and compost, all of the parasitism activity was restricted to pupae placed directly on the surface. Parasitism activity in vermiculite showed that the average number of pupae parasitized decreased with depth of substrate. These results suggest that fly pupae situated deeper in the substrate are less subjected to parasitism by *N. vitripennis* .

## Introduction

Successful parasitism by insect parasitoids is a complex process ( Brodeur and Boivin 2004 ). To maximize their fitness, parasitoid females must parasitize the optimal number of adequate hosts ( Carton et al. 1986 ). However, hosts might not be found directly in their emergence site. Therefore, the process of successful parasitism can be divided into four hierarchical steps, consisting of (1) hosthabitat location, (2) host location, (3) host acceptance, and (4) host regulation ( Vinson and Iwantsch 1980 ; Brodeur and Boivin 2004 ; Voss et al. 2009 ).

When female parasitoids are sexually mature, they leave their emergence patch to search for environmental niches that typically harbour their host, whether or not the host is actually present ( Laing 1937 ; Castelo et al. 2010 ). Hymenopteran parasitoids have developed several sensory and behavioural mechanisms to locate their hosthabitat and hosts, such as visual ( Herrebout 1969 ; Glas and Vet 1983 ), vibrational ( Lawrence 1981 ; Casas et al. 1998 ), and tactile cues ( Perez-Maluf et al. 2008 ; Voss et al. 2009 ), and semiochemicals ( Vinson 1976 ; Meiners et al. 2003 ; Rains et al. 2004 ; Fellowe et al. 2005 ; Cusumano et al. 2010 ). The odorant stimuli released by the host’s habitat are the primary stimuli that are detected by olfaction and can act as long range cues in the host location process, as they are usually produced in large amounts and are highly detectable ( Laing 1937 ; Vinson 1976 ; Voss et al. 2009 ).

Once the host’s habitat is located, female parasitoids must find the host itself. This step primarily relies on semiochemicals emitted by the host (i.e., frass, silk, etc.) ( Vinson 1976 ; Meiners et al. 2003 ; Takasu et al. 2007 ). Parasitoids also exploit the semiochemical communication system of its host such, as pheromones ( Powell and Pickett 2003 ). Several studies have shown that parasitism rates vary with fly breeding habitats ( Rueda and Axtell 1985 ; Smith and Rutz 1991 ; 1991b), substrate moisture, and light levels ( Legner 1977 ; Smith and Rutz 1991 ; Geden 1999 ). However, the depth at which the hosts are located in the substrate is another important ecological characteristic and is likely to play an important role in the parisitoid locating a host ( Geden 2002 ). Moreover, preferred depth varies among parasitoid species. Legner (1977) examined the depth at which *Muscidifurax* spp. and *Spalangia* spp. concentrated their foraging efforts and concluded that *Muscidifurax* spp. preferred to parasitize pupae near the substrate surface, whereas *Spalangia* spp. were more effective at locating buried hosts.

*Nasonia vitripennis* Walker (Hymenoptera: Pteromalidae) is a gregarious ectoparasitoid that attacks the pupae of several fly species of forensic importance, including blowflies, flesh flies, and houseflies ( Whiting 1967 ). These wasps are regularly found on carcasses ( Blanchot 1995 ; VanLaerhoven and Anderson 1999 ; Amendt et al. 2000 ; Grassberger and Frank 2004 ; Pohjoismaki et al. 2010 ), or birds’ nests ( Whiting 1967 ; King and Ellison 2005 ). *N. vitripennis* is a cosmopolitan species ( Whiting 1967 ; Darling and Werren 1990 ; Yoder et al. 1994 ) and has been intensely investigated in the subject of genetic, ecological, evolutionary, and developmental research over the last 50 years ( Darling and Werren 1990 ; Grassberger and Frank 2003 ; Steiner et al. 2006 ; Gadau et al. 2008 ). The wasps are commercially supplied and widely used for the control of the housefly *Musca domestica* L. and the stable fly *Stomoxys calcitrans* L. in dairies and poultry houses ( Mandeville et al. 1990 ; Morgan et al. 1991 ; Grassberger and Frank 2003 ) as well as on feedlots ( Floate et al. 1999 ; Grassberger and Frank 2003 ). However, little information exists on the ability of pupal parasitoids such as *N. vitripennis* to parasite necrophagous fly pupae located at various depths.

The objectives of the current study were to address the following questions about host location in relation to habitat depth: (1) Does the type of substrate affect *N. vitripennis* searching behaviour? (2) Does *N. vitripennis* adjust its searching strategy when given a choice of depths at which hosts are present?

## Materials and Methods

### Parasitoid and fly rearing

*Nasonia vitripennis* parasitoid females were collected in Belgium from pupae of *Calliphora* spp. and maintained on host pupae of *Calliphora vicina* Robineau-Desvoidy (Diptera: Calliphoridae). Male and female *N. vitripennis* were maintained together in plastic boxes (4.4 × 5.3 × 5 cm) with a 1:1 (vol/vol) honey-water solution. Parasitoid wasps and blowfly laboratory colonies ( *C. vicina* ) were reared at 23 ± 1°C with a daylight regime of 16:8 L:D and 70% RH. Male and female blowflies were maintained together in a rearing cage (55 × 60 × 48 cm) supplied with sucrose, dried milk, and water. Defrosted pork chop was used to induce blowfly oviposition and as a food source for blowfly larvae. The experiments were conducted on naïve 2–5- day-old female *N. vitripennis* and with 6-day- old pupae of *C. vicina* . The pupae of this age are the most attractive to parasitism by *N. vitripennis* (C. Frederickx, personal observation).

### Impact of the substrate types and depth on *N. vitripennis* foraging behaviour

Two types of bioassays were conducted to determine the ability of parasitoids to locate pupae at different substrate depths under choice and no choice situations.

The purpose of the first bioassay was to assess host location and parasitism by parasitoids when they were exposed to pupae buried at a single depth in each of three substrates. In this no choice bioassay, 15 fly pupae, 5–6-days old, were placed in a cylindrical plastic box (7 cm diameter, 25 cm height) in each of the three substrates at either 0 (on the surface), 1, 2, or 4 cm from the top of the substrate. The height of the substrate column was held constant at 7 cm for all burial treatments. Five naïve female parasitoids (2–5 days old) were introduced into each box, and the boxes were covered with a net and held at 23 ± 1°C and 70% RH with a daylight regime of 16:8 L:D. Pupae were removed from the plastic box after 48 hr, separated from any parasitoids present, and transferred to a Petri dish for fly and parasitoid emergence. Ten replicates per depth and per substrate were made. Ten control fly emergence was assessed by placing fly pupae in a box without parasitoids at depths of 0, 1, 2, or 4 cm from the substrate surface per substrate tested.

The second bioassay (choice assays) was conducted to evaluate the ability of the parasitoids to locate pupae presented simultaneously at a variety of substrate depths (0, 1, 2, or 4 cm from the substrate surface). Fifteen pupae (5– 6 days old) per depth level (total of 60 pupae) were placed in a Plexiglas box (24 × 17 × 9 cm) containing one of the three substrates. The height of the substrate column was held constant at 7 cm. In the substrates, the pupae depth was chosen randomly. Five naïve female parasitoids (2–5 days old) were released into the box. Ten replicates per substrate were made (total of 30 replicates). Pupae were removed from the box and transferred to a Petri Dish after 48 hr of exposure to the parasitoids, as before. Ten controls were performed with the same method but without parasitoids.

### Substrates

In order to investigate the impact of different substrates on *N. vitripennis* foraging behaviour, three substrates with distinct physical properties were selected.

The first substrate was a loam soil containing 90% loam, 7% sand, and 3% clay. The granulometry was between 0.15 mm and 0.5 mm, and the moisture content was 4%.

The second substrate was a compost containing 20% organic matter (Compo Sana®, universal compost, www.compo.com ). This substrate contained mixed peat, pearlite, silicic colloid, fertilizer containing calcium, and magnesium. The granulometry was between 0.5 mm and 1 mm, and the moisture content was 5%.

The third substrate consisted of exfoliated vermiculite (Sibli SA®, www.sibli.be ). The composition of this substrate was SIO2: 39%, MgO: 25%, AL2O3: 11%, H2O: 10%, Fe2O3: 8%, CaO: 3%, K2O: 3%, and TIO2: 1%. The granulometry was between 1 and 2 mm, and the moisture content was < 1%.

### Statistical analyses

Soil and depth preferences correspond to the number of hosts attacked. For each type of bioassay, the number of pupae parasitized was analysed by a two-way ANOVA. Statistical tests were performed with the statistical software Minitab® v15.0 ( www.minitab.com ) for Microsoft Windows® ( www.microsoft.com ). An analysis of variance, with factors being substrate and depth, was conducted. When a significant difference was observed in terms of number of pupae parasitized based on the depths or substrates, a multiple comparison of the means was carried out using the method of Newman and Keuls (α = 0.05). The data were normalized before ANOVA by an angular transformation. Control mortality of pupae was calculated for each substrate, depth, and type of bioassay. No significant differences in control mortality were observed between the three substrates and the four pupal burial treatments for each type of bioassay. Thus, these data were not used in conducting the ANOVAs. The mortality was averaged 0– 20% for the two bioassays.

## Results

### Impact of the substrate type and depth on *N. vitripennis* foraging behaviour in no choice bioassays

In the no-choice bioassay, the pupae depth significantly influenced the parasitizing rates in the three tested substrates, loam soil, compost, and vermiculite (F3,108 = 35.46, *P* < 0.001; F3,108 = 44.74, *P* < 0.001; F3,108 = 52.09, *P* < 0.001, respectively). The multiple mean comparisons by the Newman and Keuls test showed that the pupae on the surface were more often attacked and parasitized in loam soil and in compost than those placed under the soil surface level. In loam soil, 59.33% of pupae were parasitized on the surface and none under the ground ( Table 1 ). In compost substrate, 70% and 0.66 % of pupae were parasitized at 0 cm and 1 cm depth, respectively, and none were parasitized at the greater depths. When testing the vermiculite substrate, *N. vitripennis* parasitized more preferentially pupae located on the surface (88.67%), but 40.67% of pupae were also parasitized at a depth of 1 cm. Small numbers of hosts were also attacked and parasitized at depths greater than 1 cm in this substrate (7.33% and 14 % at 2 and 4 cm, respectively). The mean comparison showed that the pupae located at 2 or 4 cm were not parasitized differently in this substrate. At 0 and 1 cm (F2,108 = 7.59, *P* < 0.001; F2,108 = 19.83, *P* < 0.001, respectively), *N. vitripennis* was most effective at locating host pupae in vermiculite substrate than the other substrates. At depths of greater than 1c m, pupae were equally parasitized in each substrate (2 cm: F2,108 = 0.64, *P* = 0.53; 4 cm: F2,108 = 2.40, *P* = 0.096, respectively).

**Table 1.**
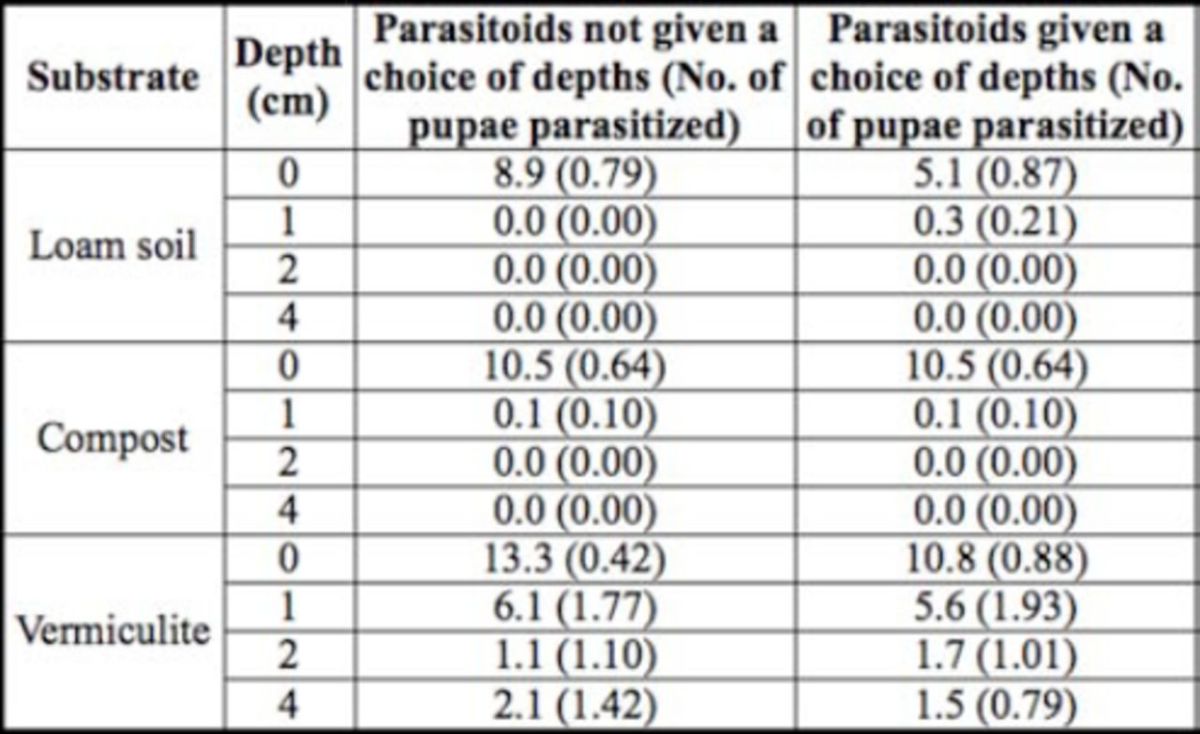
Mean number (± SEM) of pupae parasitezed by Nasonia vitripennis when house fly pupae were placed at various depths in loam soil, compost, a nd vermiculite substrate.

### Impact of the substrate type and depth on *N. vitripennis* foraging behaviour in choice bioassays

In the choice bioassay, the two-way ANOVA indicated that the depths at which the pupae were placed influenced the parasitizing rate in loam soil, compost substrate, and vermiculite substrate (F3,108 = 15.00, *P* < 0.001; F3,108 = 42.51, *P* < 0.001; F3,108 = 27.76, *P* < 0.001, respectively). The multiple mean comparisons by the Newman and Keuls test showed that the pupae on the surface were more attacked and parasitized in loam soil and in compost substrate when compared to the other pupal burial depths for both substrates. In loam soil, at depths of 0 and 1 cm, 34% and 2% of pupae were parasitized when parasitoids were given a choice of burial depth ( Table 1 ). In compost substrate, 70% of the pupae placed on the surface were attacked and parasitized. At 1 cm, only 0.66% of pupae were parasitized. Host attack was null at depths greater than 1 cm in compost substrate. For vermiculite substrate, *N. vitripennis* killed differently pupae on the surface and at 1 cm below the surface. *N. vitripennis* parasitized more preferentially pupae located on the surface, with 72% of pupae being parasitized. Moreover, *N. vitripennis* parasitized 37.33% of host pupae placed 1 cm below the surface of vermiculite. Pupae located at 2 or 4 cm were not parasitized differently in this substrate. 11.33% and 10% of pupae were parasitized at these depths, respectively. Host attacks were more frequent on the surface of compost and vermiculite substrates than on loam soil (F2,108 = 10.18, *P* < 0.001). At 1 cm below the surface (F2,108 = 17.13, *P* < 0.001), vermiculite substrate was more preferred by *N. vitripennis* to parasitize host pupae than the two others substrates. At depths of greater than 1 cm, pupae were equally parasitized in each substrate (2 cm: F2,108 = 2.17, *P* = 0.119; 4 cm: F2,108 = 2.44, *P* = 0.092).

## Discussion

Parasitoids respond to cues in ways that reflect their value and detectability in the ecological setting in which they evolved ( Morgan and Hare 1998 ). The ability of females to find the host environment is affected by the odour of pupae ( Edwards 1954 ; Edwards 1955 ; Wylie 1958 ; Vinson 1976 ; Meiners et al. 2003 ; Takasu et al. 2007 ). These odours, in contrast to the more long-range volatile chemicals of host habitat, appear to orient the parasitoid only when it is a short distance away (2–20 cm) ( Hendry et al. 1973 ; Vinson 1976 ). The substrates in which larvae of blowflies develop are ephemeral ( Gomes et al. 2005 ; Gomes and Von Zuben 2005 ; Gomes et al. 2006 ). After the exhaustion of food, larvae begin dispersing to find adequate places for pupation, a process named postfeeding larval dispersal ( Greenberg 1990 ; Gomes et al. 2005 ; Gomes and Von Zuben 2005 ; Gomes et al. 2006 ; Arnott and Turner 2008 ). Carrion flies can be divided into those that pupate in or near the food source, such as *Piophilidae* , and those that move away from the carcass to bury into the soil before pupation, such as *Calliphoridae* and *Muscidae* ( Voss et al. 2009 ). King (1997) demonstrated that host burial greatly reduces parasitism. In 1977, Legner examined the depth at which parasitoids foraged and concluded that *Muscidifurax* species ( *Muscidifurax uniraptor* Kogan & Legner and *M. zaraptor* Kogan & Legner) concentrated their efforts near the substrate surface, whereas *Spalangia* spp. ( *S. endius* Walker and *S. cameroni* Perkins) were more effective at locating buried hosts ( Legner et al. 1974 ; Legner 1977 ). Pupal parasitism by *Muscidifurax* spp. greatly decreased if hosts were located at depths ≥ 1 cm ( Floate and Spooner 2002 ; Geden 2002 ; Pitzer et al. 2011 ). In contrast, both *S. cameroni* and *S.endius* searched uniformly through a commonly used fly rearing medium and regularly located hosts at 6 cm depths in the porous, relatively loose substrate ( Legner 1977 ; King 1997 ; Geden 2002 ; Skovgard 2006 ). With *N. vitripennis* , Ullyett (1950) reported a higher incidence of parasitism in pupae located on or near the surface of a carcass than those buried in the soil. A large proportion of the pupae in birds’ nests may be parasitized by *N. vitripennis* , probably because the pupae are not buried and are in a limited habitat ( Whitehead 1933 ; Wylie 1958 ). In this study, female *N. vitripennis* parasitized more preferentially pupae located on the surface. In accordance with several studies, *N. vitripennis* is not considered to be adapted for burrowing, and buried pupae are typically beyond the reach of parasitizing females ( Altston 1920 ; Ullyett 1950 ; Whiting 1967 ). It is surprising that *N. vitripennis* has such a narrowly defined preference for pupae placed on the surface of substrates because they attack a diversity of host’s (blow fly, flesh fly, and house fly) pupae associated with cadavers, bird nestlings, and dairy farms ( Whiting 1967 ; Smith and Rutz 1991 ; Blanchot 1995 ; VanLaerhoven and Anderson 1999 ; Amendt et al. 2000 ; Grassberger and Frank 2004; King and Ellison 2005 ; Pohjoismaki et al. 2010 ). Although these experiments provided useful comparisons of different depth searching behaviour, their utility for predicting behaviour in the field is limited because of the type of substrate used (vermiculite, loam soil, and compost) and because the parasitoids were restricted to single substrate treatments in the bioassays.

Several studies have reported significant effects of habitat substrate on house fly parasitism ( Greene et al. 1989 ; Smith and Rutz 1991 ; Olbrich and King 2003 ). Like these studies, our experiments with pupae of *Calliphora vicina* placed on different substrates showed that pupae in vermiculite soil were more parasitized than those placed in the two other substrates. However, pupae in loam soil and in compost substrate were not parasitized differently. This observation was also reported in other studies ( Meyer et al. 1991 ; Seymour and Campbell 1993 ). The effect of substrate on parasitism may be due to the medium porosity, as Smith and Rutz (1991) highlighted in one of their experiments. Contrary to some publications ( Floate and Spooner, 2002 ), the present study showed an effect of medium porosity on parasitization. However, the available pore space in the soil contributes to the negative relation between soil compaction and pupation depth ( Ullyett 1950 ; Geden 2002 ; Cammack et al. 2010 ). In vermiculite, there are a lot of spaces between particles compared to the compost and loam soil. In these two last substrates, compaction was higher than vermiculite, so pore space decreases ( Babercheck 1992 ), reducing gas exchange in the soil; thus, less oxygen is available for pupae and parasitoids ( Brady and Weil 2008 ; Cammack et al. 2010 ). Larvae, therefore, might pupate closer to the soil surface where more oxygen is available. In loam soil and in compost, this compaction and thus the lack of pore space and oxygen may explain the non-parasitization of pupae by *N. vitripennis* under the ground. Moreover, in the field, pupating closer to the surface increases susceptibility to predation and parasitism ( Guillen et al. 2002 ).

In conclusion, the present study indicates that fly pupae situated deeper are less subjected to parasitism by *N. vitripennis* . Furthermore, the expression of preference in the absence of competitors suggests that *N. vitripennis* is innately restricted to foraging within specific depths. This implies that for pest species that occupy a variety of microhabitats, the successful application of biological control may depend on identifying a group of natural enemies that have complementary niches ( Smith and Rutz 1991 ). House flies are an example of such a pest, and the results of this experiments may provide a basis for recommending which parasitoids species are most likely to provide successful biological control at different types of fly-breeding sites ( Smith and Rutz 1991 ; Gadau et al. 2008 ). For successful biological control, several principal attributes of a natural enemy are important. The enemy should have (i) a general good adaptation to the environment and the host, (ii) a high rate of population increase relative to its host, (iii) a general mobility adequate for dispersal, and (iv) minimal lag effect in responding to host changes in numbers ( Huffaker and Kennett 1969 ; Skovgard 2006 ). However, a factor not involved above is the ability of released pupal parasitoids to penetrate deep into organic material for fly pupae ( Skovgard 2006 ). The female's inability to burrow into the ground, where many of its potential hosts occur, seriously limits the efficiency of the species as a biological control agent ( Wylie 1958 ). The results of our study confirm that *N. vitripennis* do not burrow in soil. Thus, this insect is not adapted as a biological control agent toward house fly pupae. However, burrowing of pupae in soil might not protect pupae from parasitism by other species of Hymenoptera that enter soil to parasitize hosts, such as *Alysia manducator* Panzer or *Spalangia cameroni* Perkins ( Legner 1977 ; King 1997 ;G eden 2002 ; Skovgard 2006 ).

A better knowledge of the foraging behaviour will assist entomological collections at crime scenes. Given the high likelihood of host parasitization on the ground by *N. vitripennis* , the appropriate search and handling protocol of pupal remnants should be conducted during the collection of entomological evidence. It is preferable to collect a great number of pupae in the ground and not on the ground surface because the rate of parasitism is more important. Moreover, pupae that have not emerged simultaneously with their cohort should be treated as potential hosts of parasitoids and reared appropriately.
